# Supplementary material for: Tracking down the molecular architecture of the synaptonemal complex by expansion microscopy
Source: Nat Commun. 2020 Jun 26;11:3222. doi: 10.1038/s41467-020-17017-7 (PMC7320163; doi:10.1038/s41467-020-17017-7)
Supplement: Supplementary file 1 — Supporting Information [file 41467_2020_17017_MOESM1_ESM.pdf]

Tracking down the molecular architecture of the synaptonemal complex by expansion microscopy

Zwettler et al.

Supplementary Table 1. Immunolabeling used in the different experiments

	Primary antibodies	Secondary antibodies	Figures
***d*STORM**	SYCP3 (guinea pig)	Al647 goat anti guinea pig	Figs. 2b,e<br>Supplementary Fig. 3
**Unexpanded SIM**	SYCP3 (rabbit)	Al568 goat anti rabbit	Figs. 2a,d
**Unexpanded RCM**	SYCP3 (mouse)	Al488 goat anti mouse	Supplementary Figs. 6 and 7
**MAP-SIM**<br>**MAP-RCM**	SYCP3 (mouse)<br>SYCE3 (rabbit)<br>SYCP1N (guinea pig)	SeTau647 goat anti mouse<br>Al568 goat anti rabbit<br>Al488 goat anti guinea pig	Figs. 2c,f<br>Figs. 3-5<br>Supplementary Figs. 1,2,6,7,10
**U-ExM SIM**	SYCP3 (mouse)<br>SYCE3 (rabbit)<br>SYCP1N (guinea pig)	SeTau647 goat anti mouse<br>Al568 goat anti rabbit<br>Al488 goat anti guinea pig	Supplementary Fig. 4
**Pro-ExM**	SYCP3 (mouse)<br>SYCP1N (rabbit)	Al488 goat anti mouse<br>Al647 goat anti rabbit	Supplementary Fig. 4

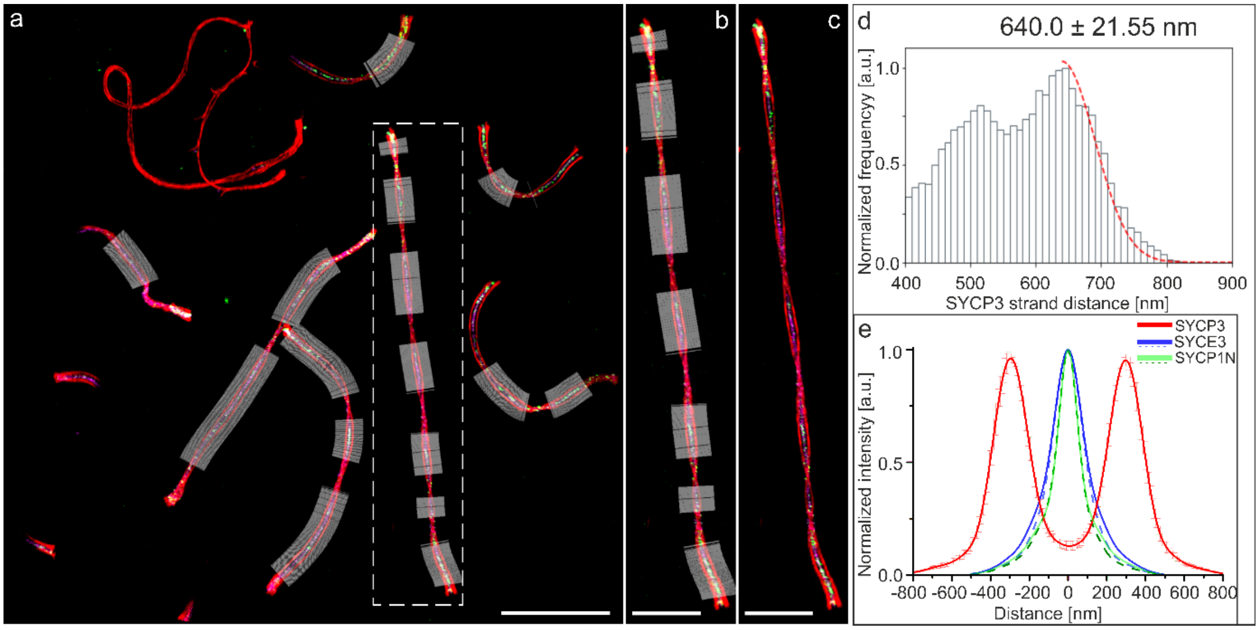

Supplementary Figure 1. **Line Profiler 2-channel mode. a**, Three-color-SIM image (maximum intensity projection) with line profiles (white) oriented along the SC at regions where SYCP3 shows a bimodal signal distribution using SYCE3 as criterion for the center of the SC. SYCP3 labeled with SeTau647 (red), SYCP1N labeled with Alexa Fluor 488 (green) and SYCE3 labeled with Alexa Fluor 568 (magenta). **b**, Magnified view of white dashed box in (a). **c**, Same as (b) without line profiles. **d**, Histogram of SYCP3 distances of 17,607 line profiles determined from 97 MAP-SIM SC images from two independent experiments. The SYCP3 distance has been determined to 640 ± 21.6 nm (SD) (see Methods for more details). **e**, Averaged intensity profiles of SYCP3 (red), SYCE3 (blue) and SYCP1N (green) of all analyzed MAP-SIM data in (d). Dashed curves show the averaged protein distribution of line profiles only set at frontal views of SYCP3. Whereas solid lines are from data along the complete SC including areas where SYCP3 shows a helical crossing. SYCP1N and SYCE3 showed a monomodal protein distribution of 161.5 ± 1.3 nm (FWHM) for SYCP1N and 229.3 ± 1.2 nm (FWHM) for SYCE3 including SYCP3 crossing point areas. Analysis of the protein distributions without SYCP3 crossing point areas resulted in FWHM of 160.2 ± 1.1 nm for SYCP1N and 207.5 ± 0.78 nm for SYCE3. Peak-to-peak distances were determined by fitting a half-normal distribution function to the histograms. Scale bar. (a) 10 µm. (b-c) 5 µm.

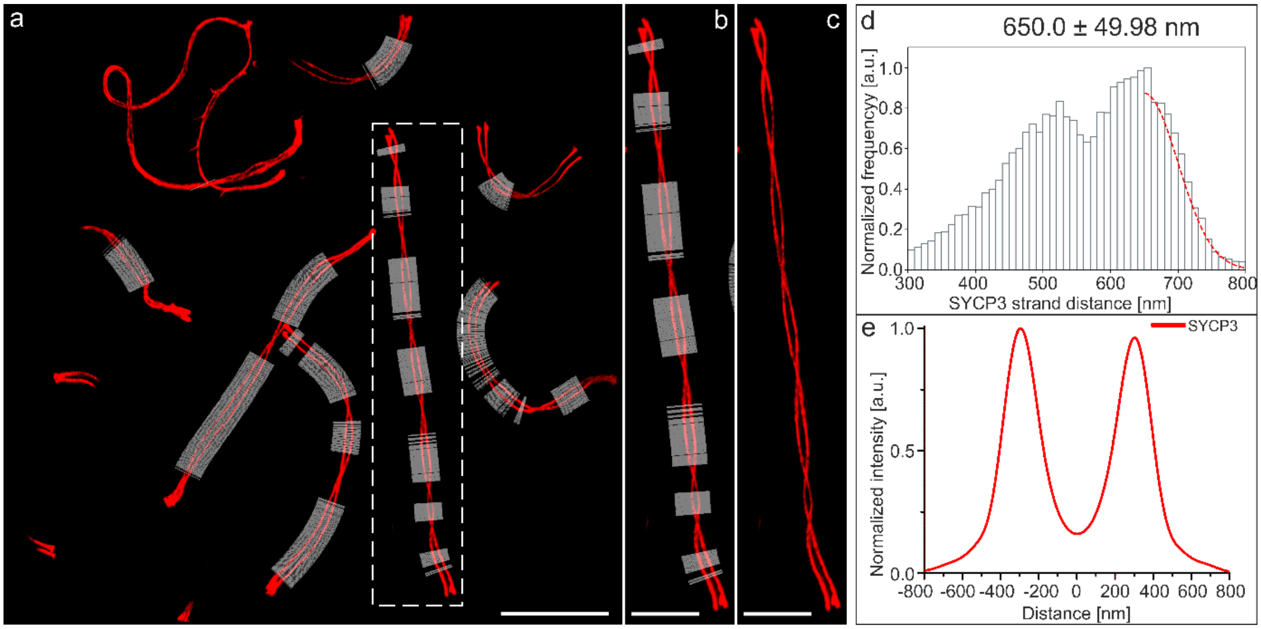

Supplementary Figure 2. **Line Profiler 1-channel mode**. **a**, Same three-color-SIM image (maximum intensity projection) as shown in Supplementary Fig. 1 but with line profiles (white) oriented along the SC with SYCP3 (red) as criterion for the center of the SC. Profiles are set at regions where a bimodal signal distribution of the protein is occurring. **b**, Magnified view of white dashed box in (a). **c**, Same as (b) with SYCP1N (green) and SYCE3 (magenta) without cross-sectional profiles. **d**, Histogram of SYCP3 distances of 24,157 line profiles determined from the same data set as analyzed in Supplementary Fig. 1. The SYCP3 distance has been determined to 650.0 ± 50.0 nm (SD) (see Methods for more details). **e**, Averaged intensity profiles of SYCP3 (red), SYCP1 N (green) and SYCE3 (magenta) of the analyzed MAP-SIM data in (d). Peak-to-peak distances were determined by fitting a half-normal distribution function to the histograms. Scale bar. (a) 10 µm. (b-c) 5 µm.

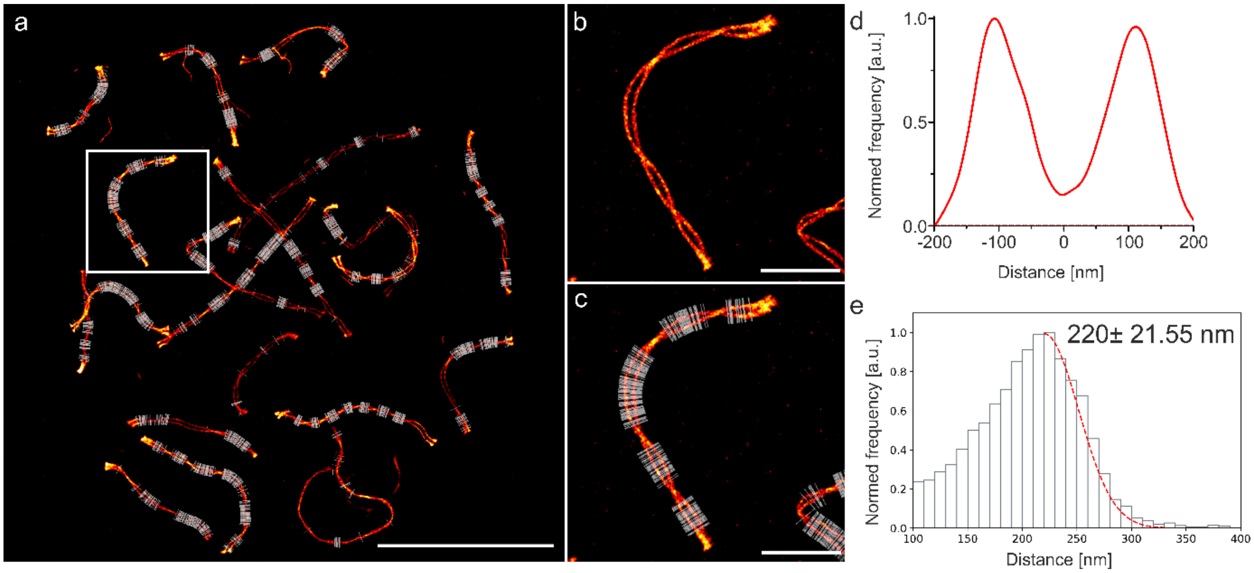

Supplementary Figure 3. ***d*STORM image and analysis of unexpanded SCs**. **a**, SYCP3 was labeled with Alexa Fluor 647 (red) with intensity line profiles (white) along the SC using SYCP3 to define the center and orientation of the complexes. **b,** Enlarged view of white box highlighted in (a) without line profiles. **c,** Same as (b) with line profiles (white) along the SYCP3 signal. **d,** Averaged intensity line profile of 7,501 line profiles analyzed in six *d*STORM images of spreaded spermatocyte cells immunolabeled with primary SYCP3 and Alexa Fluor 647 conjugated secondary antibodies. **e**, Histogram of SYCP3 strand distances resulting from line profiles shown in (a) with a mean value of 220.0 ± 21.6 nm (SD) determined by fitting a half-normal distribution function to the histograms. Scale bars, (a) 10 µm, (b-c) 2 µm.

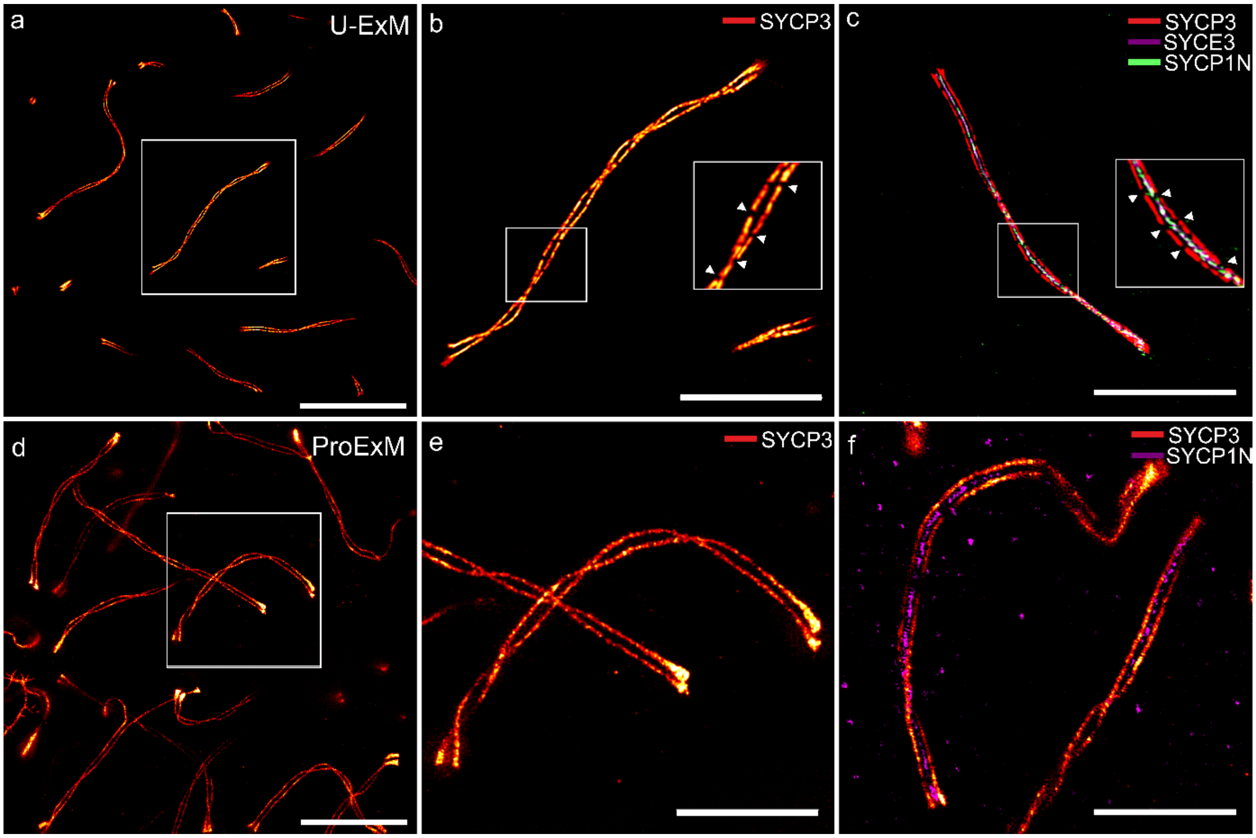

Supplementary Figure 4. **SIM images of expanded SCs**. **a**, SIM image (maximum intensity projection of a z-stack) of U-ExM expanded spermatocytes, post-expansion labeled with antibodies against SYCP3 and SeTau647 labeled secondary antibodies. **b**, Magnified view of boxed region in (**a**) with inlet showing the zoom-in of the white boxed area with arrow heads pointing at structural breaks along the SC. **c**, Multicolor SIM image (MIP) of an U-ExM treated SC immunolabeled using antibodies against SYCP3 (red, labeled with SeTau647), SYCE3 (magenta, labeled with Alexa Fluor 568) and SYCP1N (green, labeled with Alexa Fluor 488). The inlet shows the zoom in on the white boxed area with arrow heads pointing at structural breaks along the SC. **d**, SIM image (maximum intensity projection of a z-stack) of proExM expanded SCs, pre-expansion labeled for SYCP3 by immunolabeling with Alexa Fluor 488. **e**, Magnified view of boxed region in (d). **f**, Maximum intensity projection of a z-stack of a two-color SIM image of a proExM expanded SC pre-expansion labeled with Alexa Fluor 488 for SYCP3 (red) and SYCP1N labeled with labeled with Alexa Fluor 647 (magenta). Scale bars. (a) 20 µm, (b-c) 10 µm, (d) 20 µm, (e-f) 10 µm.

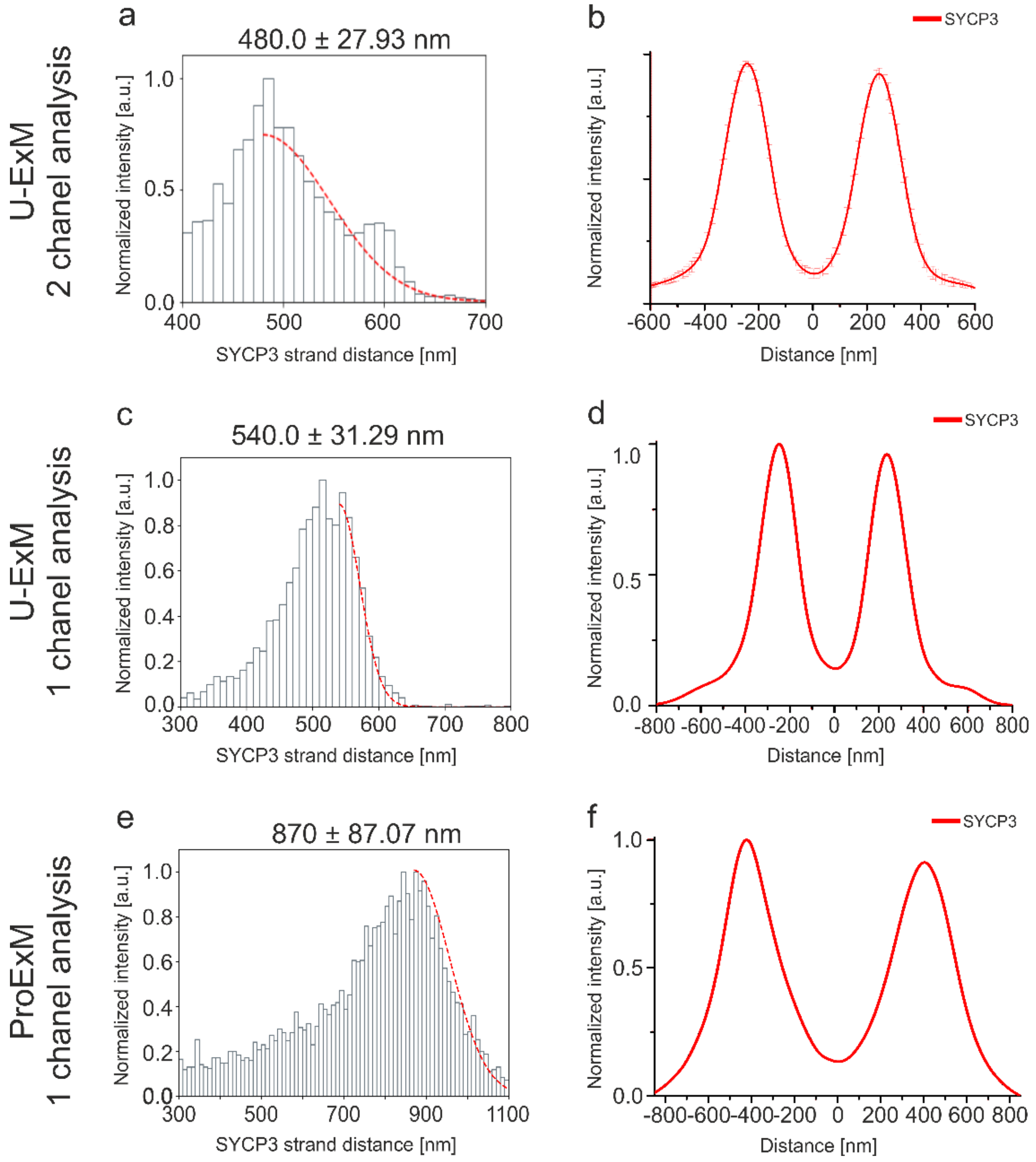

Supplementary Figure 5. Data analysis of expanded SCs. a, Histogram of SYCP3 distances determined from U-ExM experiments. The strand distance has been determined to 480 ± 27.9 nm (SD) from 24 expanded SCs in two independent expansion experiments. The SYCE3 channel was used to align the line profiles. **b,** Averaged intensity profiles of SYCP3 (red) of U-ExM data from (a). **c-d,** Same data as in (a). Here, the SYCP3 channel was used to align the line profiles. The strand distance has been determined to 540 ± 31.3 nm (SD) determined from 24 expanded SCs in two independent expansion experiments. **d,** Averaged intensity profile of SYCP3 (red) of U-ExM data analyzed in (c). **e,** Histogram of SYCP3 distances of proExM experiments. The strand distance has been determined to 870 ± 87.1 nm (SD) measured along 50 expanded SCs. The SYCP3 channel was used to align the line profiles. **f,** Averaged intensity profiles of SYCP3 (red) of proExM data from (e). All strand distance valueswere determined by fitting a half-normal distribution function to the histograms (red dashed curves).

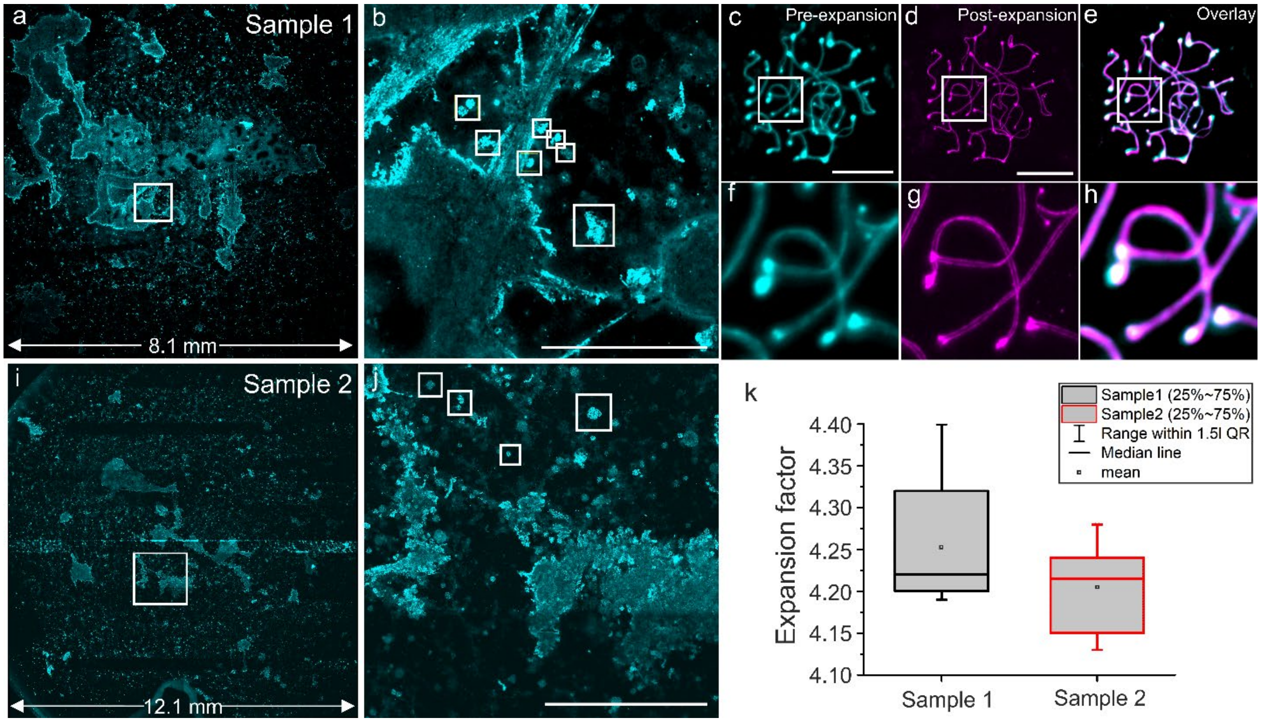

Supplementary Figure 6. Determination of MAP-SIM expansion factor. a,i, Stitched re-scan confocal microscopy (RCM)[1,2] images of two 18 mm coverslips (sample 1 and sample 2) with spreaded spermatocytes immunostained with SYCP3 primary and Alexa Fluor 488 conjugated secondary antibodies acquired using an 10x objective. **b, j,** Zoom in on highlighted regions in (a) and (**i**) with white box marked cells used to determine the MAP-SIM expansion factor. **c,d,** Pre-expansion RCM image (Maximum intensity projection, MIP) of SYCP3 labeled with Alexa Fluor 488 shown in cyan (c) and post-expansion RCM image (MIP) of the same cell labeled post-expansions with antibodies against SYCP3 and secondary SeTau647 antibodies (d). **e,** Overlay of pre- and post-expansion images shown in (**c-d**) using a rigid image registration. **f-h,** Zoom-in on highlighted regions in (c-e). **k,** Distribution of expansion factors determined from similarity transformations as shown in (c-h) from 7 cells in sample 1 (a) and 6 cells in sample 2 (i). Scale bars, (b) 400 µm, (c) 10 µm, (d) 40 µm, (j) 800 µm.

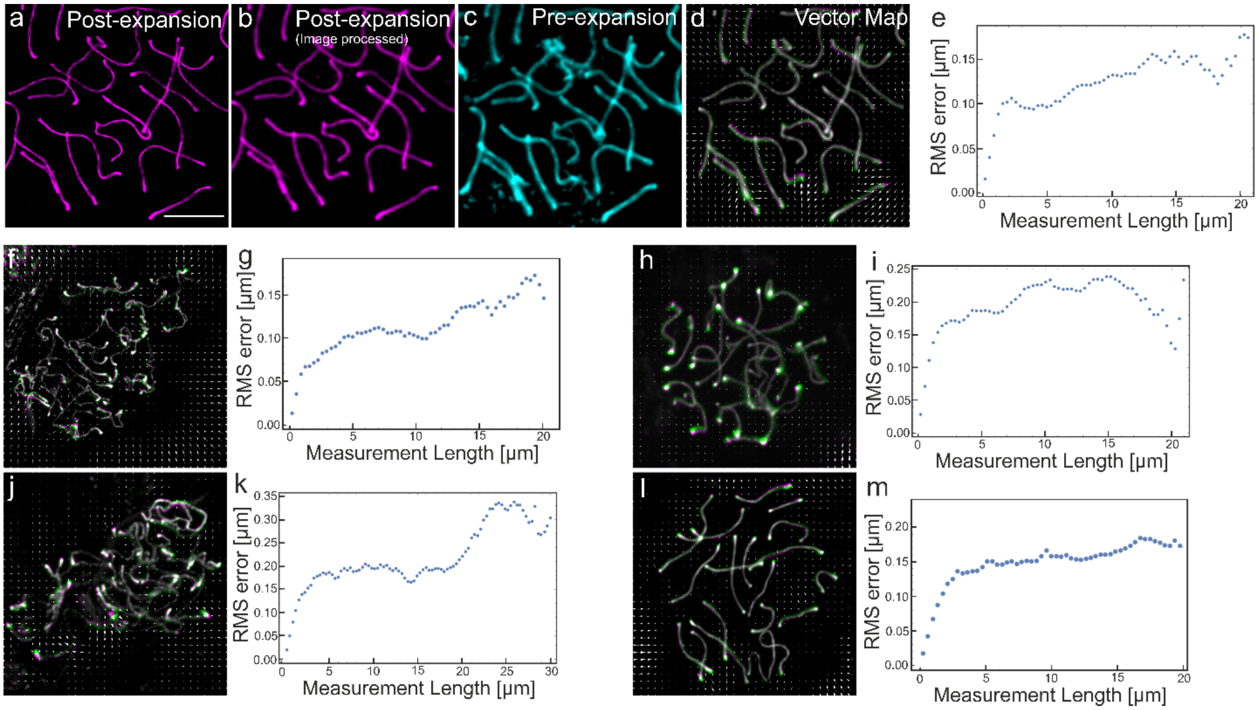

Supplementary Figure 7. Comparison of pre- and post-expansion SC images. a, Expanded MAP-SIM image (MIP) of SYCP3 labeled post-expansion with SeTau647. **b,** Same image as in (a) blurred with a Gaussian for distortion analysis. **c,** Pre-expansion image of the same cell as in (a) labeled with SYCP3 and Alexa Fluor 488 labeled secondary antibodies. **d,** Overlay of pre- (green) and post- (blue) expansion image shown in (a-b) with deformation vector map determined by a non-rigid transformation (B-Spline). **e,** RMS (root-mean-squared) error vs length distortion analysis of the vector map shown in (e). **f-m**, Deformation vector fields of pre- (green) and post- (blue) expansion re-scan confocal microscopy (RCM)[1,2] images with corresponding RMS error analyses. Scale bars, (a) 20 µm.

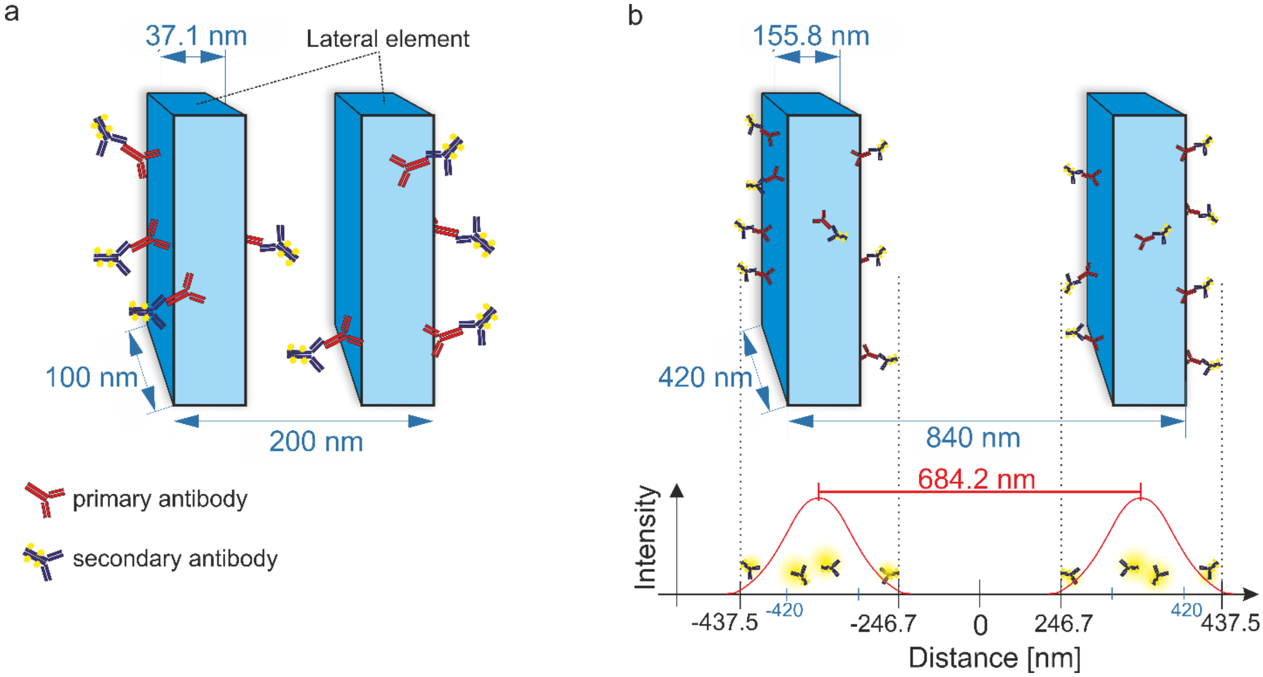

Supplementary Figure 8. Narrowing of the peak-to-peak distance of post-expansion immunolabeled SCs (SYCP3). a, Schematic illustration of indirect immunolabeling of the (unexpanded) lateral elements (blue) using primary (red) and fluorophore (yellow) conjugated secondary (dark blue) IgG antibodies. The lateral elements exhibit a width between roughly 40-50 nm in electron micrography and electron microscopy tomography (37.1 nm in murine spermatocytes)[3]. The distance of the central region, which equals the distance between the inner edges of the lateral elements, has been determined to be approximately 100 nm in a variety of organisms[4,5]. Therefore, the synaptonemal complex has a rough width of 200 nm (at a depth of 100 nm). In *d*STORM acquisitions, the peak-to-peak distance between the two strands of the lateral element protein SYCP3 has been measured to 220 nm (the distance between the lateral elements plus two antibody-antibody complexes, one on either side). The broadening effect of one antibody-antibody complex has been determined to ~17.5 nm by electron microscopy of immunolabeled microtubules[6]. We hypothesize that primary and secondary antibodies label mainly accessible SYCP3 proteins in exterior areas in unexpanded SCs because the epitopes in the center are covered by cohesion components associated with the lateral elements. Upon ~4x (Supplementary Fig. 6) expansion the SYCP3 signals are separated ~4 × 220 nm corresponding to ~ 880 nm which is in agreement with distances determined when expanding samples according the proExM protocol. **b,** Schematic representation of the lateral elements of a MAP-SIM expanded synaptonemal complex labeled post-expansion by indirect immunofluorescence. At an expansion factor of 4.2x, the distance between the two outer edges of the lateral elements should be 840 nm based on the 200 nm width of the SC determined by EM. In post-expansion expansion approaches, the antibodies are not part of the primary expansion. Due to the improved epitope accessibility of post-labeling approaches, it is likely that a higher number of epitopes at the inner edge of the lateral element towards the center of the SC are labeled. As a consequence, the maximum intensity of a SYCP3 strand can be assigned towards the center of the lateral element due to equal labeling of the inner and the outer edge of the LE. In this case, the peak-to-peak distance between the lateral element protein SYCP3 would be ~684 nm. In contrast, pre-expansion immunolabeled ~4x expanded lateral elements exhibit a larger linkage error and substantial broadening because central epitopes are not labeled.

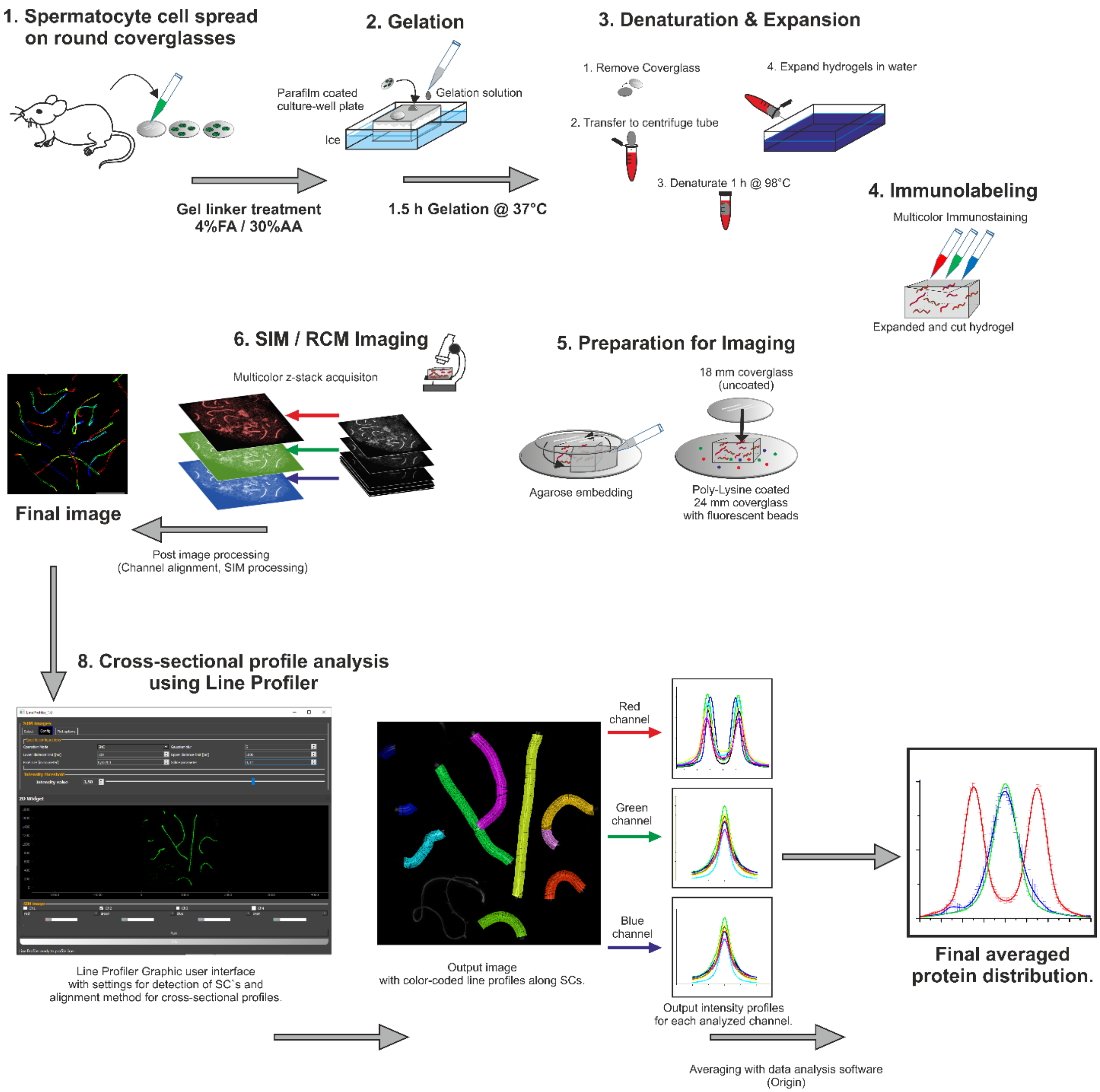

Supplementary Figure 9. Workflow of MAP-SIM expansion on SCs. 1, First, spermatocytes are extracted from mice and then spread on round 18 mm coverslips. **2,** After gel linker treatment, cells are gelated on a parafilm coated culture-well on ice. **3,** Hydrogels are then removed carefully from the cover glass and placed into pre-heated denaturation buffer. After 1 hour of denaturation gels are placed into water for expansion. **4,** Samples are immunolabeled successively with primary and secondary antibodies. **5,** Immunolabeled gels are then immobilized on Poly-Lysine coated coverslips and additionally embedded with agarose to prevent drift during imaging. **6**, Samples can then be imaged with SIM or another available imaging technique. After image acquisition post-processing results in the final images (**7**) of SCs. **8,** Images are then further analyzed using the Line Profiler software enabling analysis of protein distributions in several channels. After averaging of cross-sectional profiles a final averaged protein distribution curve is generated.

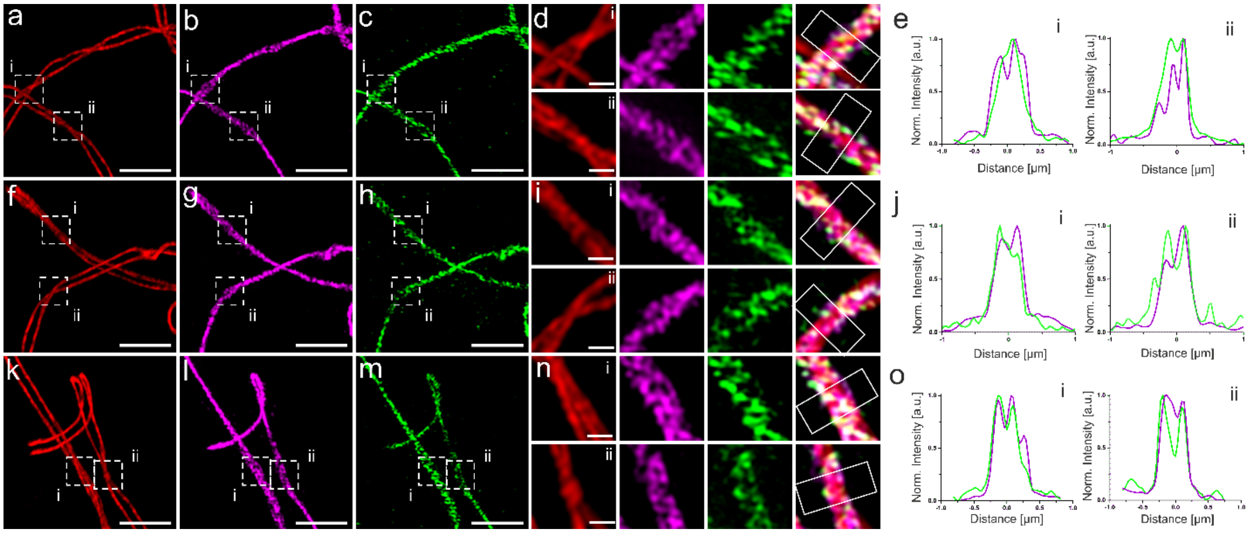

Supplementary Figure 10. Multimodal distribution of SYCP1N and SYCE3. **a-c, f-h** and **k-m** MAP-SIM images (MIP) of spread spermatocytes immunostained with antibodies against SYCP3 (red), SYCP1N (green) and SYCE3 (magenta). **d,i,n,** Single channels of white dashed boxed regions (i) and (ii) in (a-c), (f-h) and (k-m) showing the SYCP3 signal in red, SYCP1N signal in magenta and SYCE3 signal in green as well as the corresponding overlay of all three channels with white marked regions used for cross-sectional profiles. **e,j,o** Normalized intensity profiles of white boxed regions (i) and (ii) highlighted in overlay images in (d), (i) and (n) showing the multimodal distribution of the SYCP1N signal in magenta and the SYCE3 signal in green. Scale bars, (a-c, f-h, k-m) 3 µm, (d,i,n) 500 nm.

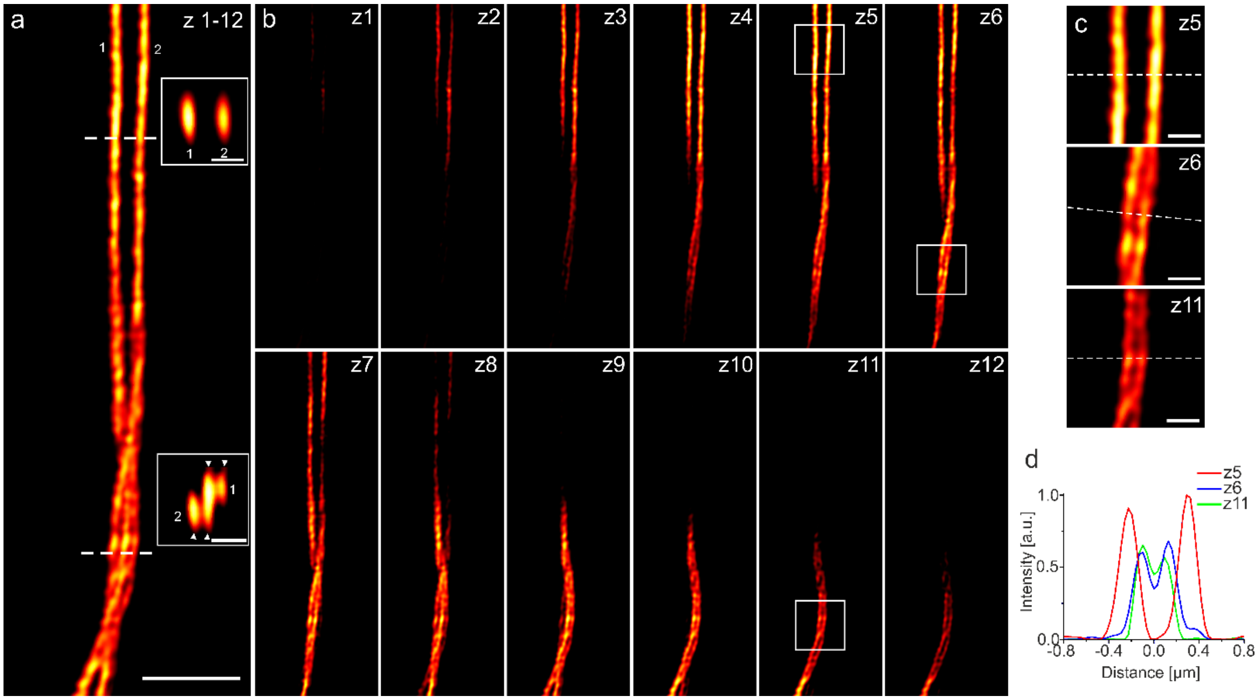

Supplementary Figure 11. Splitting of the lateral element protein SYCP3 in subLEs. a, Maximum intensity projection of 12 SIM-images (z1-12) recorded with 110 nm z-steps showing SYCP3 labeled with SeTau647 post-expansion. The image shows areas of the SC in frontal views where the lateral element can be observed as single strands (strand 1 and 2) and areas where the SC turns in side view and a splitting of the strands can be observed. The lower inlet shows the *xz* view of the white dashed line in (a) where the splitting of the strands 1 and 2 is marked with arrows. **b,** Single slices (z1-z12) of the z-stack shown in (a). **c,** Zoom in on highlighted regions in z-slice z5, z6 and z11 shown in (b) showing the lateral element in frontal view (z5) and side views (z6 and z11) where a splitting of the strands 1 (z11) and 2 (z6) can be observed. **d,** Cross-sectional profiles of the white dashed lines marked in (**c**) in the respective z-slices z5 (red), z6 (green) and z11 (blue). Note the splitting of the strands which is expressed by the bimodal distribution of the profiles z6 and z11 and the decay of the signal intensity. Scale bars, (a) 2µm.

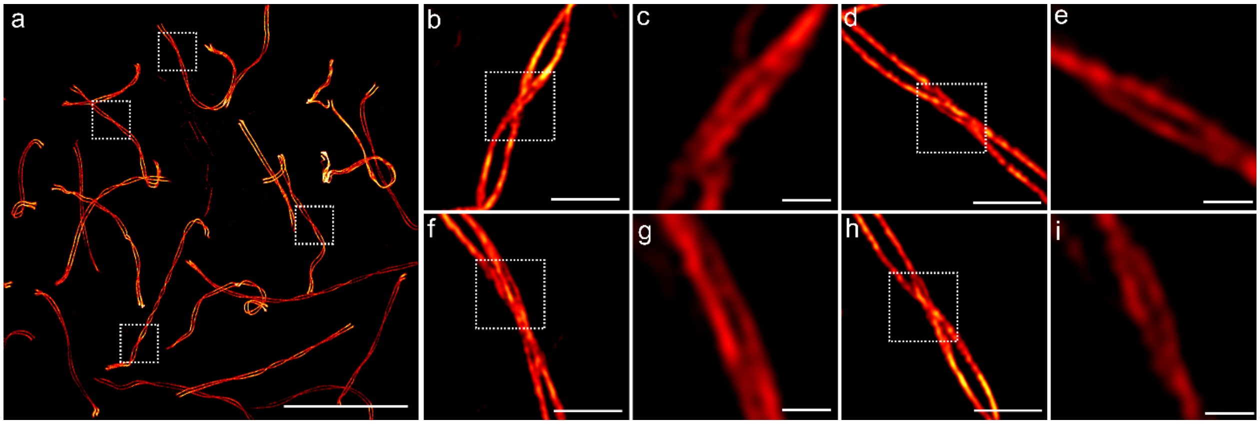

Supplementary Figure 12. Splitting of the lateral element protein SYCP3 at helix crossing points. a, Maximum intensity projection of a SIM z-stack showing SYCP3 labeled with SeTau647. **b, d, f, h,** Zoom-in on highlighted regions in (a). **c, e, g, i,** Single z-slices of the white boxed regions in (b, d, f, h) showing the splitting of the SYCP3 signal. Scale bars, (a) 20 µm, (b,d,f,h) 2 µm, (c,e,g,i) 500 nm.

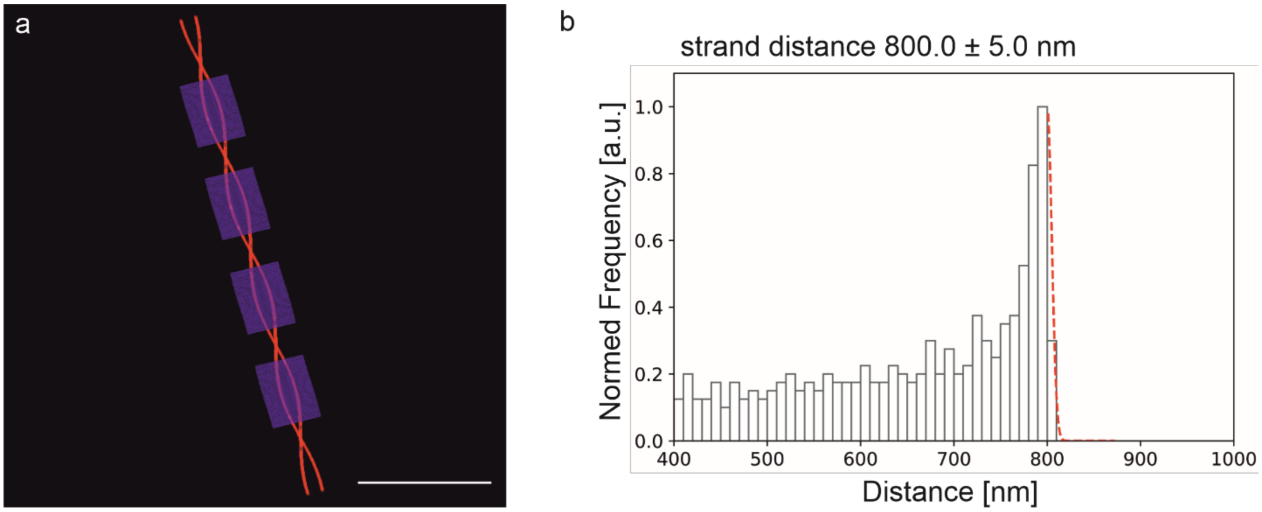

Supplementary Figure 13. Evaluation of artificial helix data with Line Profiler. a, Artificial helix data (red) created with a maximum strand distance of 800 nm rendered in 32.24 nm pixel size and profiles taken by Line Profiler (blue). The software might introduce a bias due to the fact that the profiles are not solely taken at the maximum peak to peak distance. **b,** A right sided half norm fit to the strand distances shows a maximum peak to peak distance of 800 ± 5.0 nm. This proves that our evaluation method is not biased by the decreasing distances of the helix structure. Scale bar, (a) 7 µm.

Supplementary References

1. De Luca, G. M. R. *et al*. Re-scan confocal microscopy: scanning twice for better resolution. *Biomed. Opt. Express* **4**, 2644-2656 (2013).
2. De Luca, G. M. R. *et al.* Configurations of the Re-scan Confocal Microscope (RCM) for biomedical applications. *J.l Microsc.* **266,** 166–177 (2017).
3. Spindler, M. C., Filbeck, S., Stigloher, C., & Benavente, R. Quantitative basis of meiotic chromosome synapsis analyzed by electron tomography. *Sci. Reports* **9**, 16102 (2019).
4. Ortiz, R., Kouznetsova, A., Echeverría-Martínez, O. M., Vázquez-Nin, G. H., & Hernández-Hernández, A. The width of the lateral element of the synaptonemal complex is determined by a multilayered organization of its components. *Experimental Cell Res.* **344**, 22-29 (2016).
5. Westergaard, M., & von Wettstein, D. The synaptonemal complex. *Annu. Rev. Genet*. **6**, 71-110 (1972).
6. Weber, K., Rathke, P. C. & Osborn, M. Cytoplasmic microtubular images in glutaraldehyde-fixed tissue culture cells by electron microscopy and by immunofluorescence microscopy. *Proc. Natl. Acad. Sci.* **75,** 1820–1824 (1978).
