# Supplementary material for: Tracking down the molecular architecture of the synaptonemal complex by expansion microscopy
Source: Nat Commun. 2020 Jun 26;11:3222. doi: 10.1038/s41467-020-17017-7 (PMC7320163; doi:10.1038/s41467-020-17017-7)
Supplement: Supplementary file 3 — Description of Additional Supplementary Files [file 41467_2020_17017_MOESM3_ESM.pdf]

Description of Additional Supplementary Files

File Name: Supplementary Movie 1

Description: 3D-Multicolor MAP-SIM of SYCP3, SYCP1 N-terminus, and SYCE3. Expansion microscopy (MAP) of synaptonemal complex (SC) proteins imaged with structured illumination microscopy (SIM). SYCP3 of the lateral element labeled with Setau647 (red), the N-terminus of transverse filament protein SYCP1 labeled with Alexa 488 (green) and SYCE3 of the central element labeled with Alexa 568 (magenta) on a nuclear spreading shown in pachynema. The xy pair can be distinguished by the short synapsed pseudoautosomal region indicated by the presence of all three SC proteins and the larger unsynapsed parts of the x and the y pair that are only associated with SYCP3. The movie sequence shows the progression through the acquired z-stack. SYCP3, SYCP1N and SYCE3 of the triple immunolocalization are further shown separately to provide better visibility of details of the SC's molecular architecture revealed by MAP-SIM. Note, e.g., that zoomed-in views of the lateral view sections suggest a complex architecture of the central element where SYCE3 and the SYCP1 N-terminus reside.

File Name: Supplementary Movie 2

Description: Structural details of the SC lateral element revealed by MAPSIM of SYCP3. Movie sequence showing the expanded lateral element protein SYCP3 (red, labeled with Setau647) of two SCs in pachynema. Note the fraying of the SYCP3 signal at both ends and the occasional bifurcation of the signal along the length of the SC that is in agreement with EM findings of sub-lateral elements (subLEs) in murine spreadings.
