# Supplementary material for: Tracking down the molecular architecture of the synaptonemal complex by expansion microscopy
Source: Nat Commun. 2020 Jun 26;11:3222. doi: 10.1038/s41467-020-17017-7 (PMC7320163; doi:10.1038/s41467-020-17017-7)
Supplement: Supplementary file 6 — Reporting Summary [file 41467_2020_17017_MOESM6_ESM.pdf]

nature research

Corresponding author(s): Markus Sauer

Last updated by author(s): May 21, 2020

Reporting Summary

Nature Research wishes to improve the reproducibility of the work that we publish. This form provides structure for consistency and transparency in reporting. For further information on Nature Research policies, see Authors & Referees and the Editorial Policy Checklist .

Statistics

For all statistical analyses, confirm that the following items are present in the figure legend, table legend, main text, or Methods section.

n/a	Confirmed	
☐	☒	The exact sample size (*n*) for each experimental group/condition, given as a discrete number and unit of measurement
☐	☒	A statement on whether measurements were taken from distinct samples or whether the same sample was measured repeatedly
☐	☒	The statistical test(s) used AND whether they are one- or two-sided<br>*Only common tests should be described solely by name; describe more complex techniques in the Methods section.*
☒	☐	A description of all covariates tested
☒	☐	A description of any assumptions or corrections, such as tests of normality and adjustment for multiple comparisons
☐	☒	A full description of the statistical parameters including central tendency (e.g. means) or other basic estimates (e.g. regression coefficient) AND variation (e.g. standard deviation) or associated estimates of uncertainty (e.g. confidence intervals)
☒	☐	For null hypothesis testing, the test statistic (e.g. *F*, *t*, *r*) with confidence intervals, effect sizes, degrees of freedom and *P* value noted<br>*Give P values as exact values whenever suitable.*
☒	☐	For Bayesian analysis, information on the choice of priors and Markov chain Monte Carlo settings
☒	☐	For hierarchical and complex designs, identification of the appropriate level for tests and full reporting of outcomes
☒	☐	Estimates of effect sizes (e.g. Cohen's *d*, Pearson's *r*), indicating how they were calculated

Our web collection on statistics for biologists contains articles on many of the points above.

Software and code

Policy information about availability of computer code

Data collection	NIS Elements, Zeiss ZEN black
Data analysis	Zeiss ZEN; Wolfram Mathematica 11.2; Fiji; Origin2019b; Phyton; LineProfiler

For manuscripts utilizing custom algorithms or software that are central to the research but not yet described in published literature, software must be made available to editors/reviewers. We strongly encourage code deposition in a community repository (e.g. GitHub). See the Nature Research guidelines for submitting code & software for further information.

Data

Policy information about availability of data

All manuscripts must include a data availability statement. This statement should provide the following information, where applicable:

- Accession codes, unique identifiers, or web links for publicly available datasets
- A list of figures that have associated raw data
- A description of any restrictions on data availability

The datasets generated during and/or analysed during the current study are available from the corresponding author on reasonable request.

Field-specific reporting

Please select the one below that is the best fit for your research. If you are not sure, read the appropriate sections before making your selection.

☒ Life sciences ☐ Behavioural & social sciences ☐ Ecological, evolutionary & environmental sciences

For a reference copy of the document with all sections, see nature.com/documents/nr-reporting-summary-flat.pdf

Life sciences study design

All studies must disclose on these points even when the disclosure is negative.

Sample size	Sample sizes were determined based on the estimates from preliminary experiments and similar studies in the previous manuscripts so that reasonable statistical analyses could be conducted.
Data exclusions	No data was excluded.
Replication	Experimental findings reported in this manuscript were reliably reproduced.
Randomization	Not relevant to the study.
Blinding	Blinding was not relevant to the study.

Reporting for specific materials, systems and methods

We require information from authors about some types of materials, experimental systems and methods used in many studies. Here, indicate whether each material, system or method listed is relevant to your study. If you are not sure if a list item applies to your research, read the appropriate section before selecting a response.

Materials & experimental systems

n/a	Involved in the study	
☐	☒	Antibodies
☒	☐	Eukaryotic cell lines
☒	☐	Palaeontology
☐	☒	Animals and other organisms
☒	☐	Human research participants
☒	☐	Clinical data

Methods

n/a	Involved in the study	
☒	☐	ChIP-seq
☒	☐	Flow cytometry
☒	☐	MRI-based neuroimaging

Antibodies

Antibodies used	Guinea pig and rabbit anti-SYCP1 (N-terminal amino acids 1-124), guinea pig anti-SYCP3 (N-terminal amino acids 27-38) and rabbit anti-SYCE3 (full length protein) were generated by Seqlab through immunizing the host with the respective peptides and affinity purified before use. Rabbit anti-SYCP3 (NB300-232; derived against the C-terminus) was purchased from Novus Biologicals and mouse anti-SYCP3 (ab97672; full length protein) was purchased from Abcam. Al647 goat anti-guinea pig IgG (H +L), highly cross-absorbed (A-21450) Invitrogen (ThermoFisher); goat anti-rabbit IgG (H+L) highly cross-adsorbed Alexa Fluor 568 (A-11036) was purchased from ThermoFisher; Alexa 488 conjugated AffiPure F(ab')2 goat anti-guinea pig IgG (H+L) was purchased from Dianova (106-546-003). SeTau647 NHS (K9-4149; SETA Biomedicals) was conjugated to F(ab')2 of goat anti-mouse IgG (SA-10225; ThermoFisher); F(ab')2 goat anti-rabbit IgG (H+L) cross-adsorbed Alexa Fluor 647 (A-21246) and F(ab')2 goat anti-mouse IgG (H+L) cross-adsorbed Alexa Fluor 488 (A-11017) were purchased from ThermoFisher. Supplementary Table 1 summarizes the immunolabeling used for the different experiments.
Validation	The antibodies were validated in several single, double and triple immunofluorescence assays or were validated by the respective vendors. The antibodies are specific for the synaptonemal complex of meiotic cells.

Animals and other organisms

Policy information about studies involving animals; ARRIVE guidelines recommended for reporting animal research

Laboratory animals	C57BL/6J wildtype male mice between the ages of 14 days and 12 weeks.
Wild animals	The study did not involve wild animals.
Field-collected samples	The study did not involve field collected samples.
Ethics oversight	Animal care and experiments were conducted in accordance with the guidelines provided by the German Animal Welfare Act (German Ministry of Agriculture, Health and Economic Cooperation). Animal housing, breeding and experimental protocols were approved by the regulatory agency of the city of Wuerzburg (Reference 821-8760.00-10/90; according to §11/1 No.1 of the German Animal Welfare Act) and carried out following strict guidelines to ensure careful, consistent and ethical handling of mice.

Note that full information on the approval of the study protocol must also be provided in the manuscript.
